# A Questionnaire-Based Study to Assess Knowledge and Awareness Regarding Cheiloscopy as a Forensic Odontology Diagnostic Tool Among Dental Professionals

**DOI:** 10.7759/cureus.31188

**Published:** 2022-11-07

**Authors:** Nishath Sayed Abdul, Sahar Zabin Alotaibi, Fatimah Abdullah Almughalliq, Maha Dafer Alamri, Reem Ali Alshahrani, Abdulrahman Ibrahim Almujalli

**Affiliations:** 1 Oral and Maxillofacial Surgery and Diagnostic Sciences, College of Dentistry, Riyadh Elm University, Riyadh, SAU; 2 College of Medicine, King Saud Bin Abdulaziz University for Health Sciences, Riyadh, SAU; 3 Dentistry, Imam Abdulrahman Bin Faisal University, Dammam, SAU; 4 Dentistry, King Khalid University, Abha, SAU; 5 Dentistry, Riyadh Elm University, Riyadh, SAU

**Keywords:** questionnaire, forensic odontology, dental professionals, : cheiloscopy, awareness, lip print, cheiloscopy

## Abstract

*Background*: The lips are covered with grooves and wrinkles, which form a characteristic pattern called a "lip print. The study of lip prints is called cheiloscopy. Searching for lip prints in the crime scene investigation helps in personnel identification and establishment of the true nature of the crime.

*Objective*: This study aimed to assess the knowledge and awareness of cheiloscopy among dental undergraduates, postgraduate students, and general dental practitioners.

*Materials and methods: *This cross-sectional observational, descriptive, survey-based study was conducted among 320 dental professionals, which included undergraduates, graduates, postgraduate dental students, and general dental practitioners aged between 18 and 32 years. A self-administered structured questionnaire written in English and Arabic was distributed to all willing participants. The questionnaire included knowledge and awareness-based questions along with demographic details of the participants. The Chi-square and Fisher's exact tests were applied to find out the association between the characteristics of the study participants and their knowledge and awareness of forensic odontology. A p-value of 0.05 was considered significant for all the statistical tests using IBM Corp. Released 2017. IBM SPSS Statistics for Windows, Version 25.0. Armonk, NY: IBM Corp.

*Results*: A total of 320 dental professionals completed the survey. The majority of participants (55.3%) were males (and 14.4% were females) between the ages of 23 and 27. Most of the participants were general dental practitioners (36.9%), followed by undergraduates (26.3%), graduates (8.8%), and postgraduates (18.1%). Cheiloscopy, the study of lip prints, was known to 36.6% of the participants. Whereas the majority of the participants (63.4%) were not aware of it. Postgraduate (46.7%) students had more knowledge as compared to undergraduates, graduates, and general dental practitioners. About 81.6% of the participants were not aware of the classification of lip prints by Tsuchihashi and Suzuki.

*Conclusion*:Overall, there was a lack of knowledge and awareness of cheiloscopy among all study participants, although they had good knowledge of forensic odontology. Compared to undergraduates and graduates, postgraduate dentistry students showed a greater level of cheiloscopy knowledge and awareness. Comparatively to students, general dentists, however, lacked understanding and awareness of cheiloscopy. This condition, however, can be improved if necessary steps are taken to make forensic odontology a part of the dental curriculum in Saudi Arabia.

## Introduction

The study of lip prints is known as cheiloscopy. The word cheiloscopy is derived from the Greek words "cheilo", meaning lips, and "skope-in" meaning to see [1]. The forensic investigation tool that deals with the identification of human beings and their gender is based on lip prints [2]. Forensic odontology has played a key role in the identification of people in mass disasters. The different methods used in forensic odontology include rugoscopy, cheiloscopy, bite marks, tooth prints, radiographs, photographic studies, and molecular methods. It was first identified by anthropologist R. Fischer in 1902 and described as the biological phenomenon of the system of furrows on the red part of human lips. Edmond Locard first recommended lip prints for use in personal identification and criminalization. In 1950, Synder [3] was the first person to suggest the idea of using cheiloscopy for personnel identification. From 1968-1971, two Japanese scientists, Yasuo Tsuchihashi and Kazuo Suzuki, in their studies, revealed that the arrangement of lines on the red part of human lips in an individual is unique and is not similar between two individuals [4]. They proposed a classification in 1970 based on this theory, and it was popularly known as Tsuchihashi’s classification, which classified the natural lip marks into four types. Although forensic odontology has made significant advances in the science of identifying victims and criminals through various analysis methods, one such method that is still underutilized is the study of lip prints known as cheiloscopy.

Only 26.67% of fourth-year dental students in a study by Bano and Prabhu [5] of dental undergraduate students in India reported having knowledge of cheiloscopy, and 32% of them reported having knowledge of the lip print classification described by Tsuchihashi and Suzuki as opposed to third-year students (24%). A previous study reported from Saudi Arabia includes the study conducted [6] about the morphological patterns of lip prints for gender identification (which included modified Renaud’s classification) among 13 identical twins and 19 families, which concluded by reporting that the lip prints were unique for each individual and even in twins and family relatives. The most prevalent patterns identified in their study were full bifurcation and other patterns, which were found in 68.7% of females and 42.7% of males.

In other studies, among Saudi dentists [7], about 72% agreed that lip prints are unique for each individual, and a study [8] among dental students in Riyadh, Saudi Arabia, reported that only 20% of all dental students knew about lip prints. Studies relevant to Saudi Arabia have a significant gap in the literature when it comes to cheiloscopy specifics. Only a few research have been published so far on the understanding and awareness of cheiloscopy among dental practitioners in Saudi Arabia. The main purpose of the current study was to therefore close this knowledge gap and evaluate dental professionals' knowledge and awareness of cheiloscopy across Saudi Arabia.

## Materials and methods

A quantitative, observational, online survey-based study (using a specific classification of lip prints as given by Tsuchihashi and Suzuki) was conducted among 320 undergraduate, graduate, and postgraduate dental students and general dental practitioners that were employed in one of the four institutions that were willing to be a part of our study: Riyadh Elm University (REU), Riyadh; King Saud Bin Abdulaziz University for Health Sciences (KSAU-HS), Riyadh; King Khalid University (KKU), Abha; and Imam Abdulrahman Bin Faisal University (IAFU), Dammam, Saudi Arabia. This sample size was agreed upon after using the Fisher formula for sample size calculation, with the formula being: sample size= Z2P (1-P)/ D2, where Z = coefficient of Z statistics obtained from a standard normal distribution, P = Proportion (in %) Q = 1 − P, and D = sample error tolerated (in %). 

Hence, using a prevalence rate of 8.6% [7-8] at a confidence limit of 95% (D = 5%) and Z of 3.58, the minimum sample size (N) is calculated as N = 3.582 X 8.6 (100-8.6)/ 52 hence N = 403. As a result, the study's minimum sample size (N) was to be kept around 400 individuals, which should have sufficed the needs of our study.

In the present study, 320 dental students were included, which included 84 undergraduates, 60 graduates, 58 postgraduate dental students, and 118 general dental practitioners who were practicing in clinics, hospitals, and other dental establishments. Dental students from four different universities in Saudi Arabia were included. There are 90 students from Riyadh Elm University (REU), Riyadh; 65 students from King Saud Bin Abdulaziz University for Health Sciences (KSAU-HS), Riyadh; 70 from King Khalid University (KKU), Abha; and 95 from Imam Abdulrahman Bin Faisal University (IAFU), Dammam, Saudi Arabia. Both male and female dental students, aged between 18 and 32 years, participated in this study. The study period was from June 2022 to August 2022.

The inclusion criteria for our investigation were that we included only those dental students and professionals who were willing to participate, and in terms of the exclusion criterion, those who were not willing to participate or had submitted the questionnaire with incomplete data were rejected from the domains of the study. The study participants were informed about the purpose and objective of the research, and informed consent was obtained. The ethical clearance was obtained from the Institutional Review Board of REU with IRB approval number "SRP/2022/101/760/720."

A structured and close-ended questionnaire written in English and Arabic (for convenience considering the native tongue of the region of our study, which does not violate the validity of the questionnaire in any manner) was distributed through an online survey (made using Google forms) to 320 dental professionals. Both males and females, aged between 18 and 32 years, were included. Twenty-four closed-ended, yes/no questions were included in each of the questionnaire's three sections. The first section included four items on the details of the socio-demographic data, such as age, gender, education level, and universities of dental professionals. The second section included 15 items that assessed the knowledge-based questions on forensic odontology (FO) and cheiloscopy. The third section included five items that assessed awareness-based questions on cheiloscopy. The survey questionnaire used was modified and adapted from the previously validated questionnaires [4,7,8]. Table 1, as mentioned below, lists the specifics of the questionnaire as well as the criteria for scoring.

**Table 1:** Knowledge of forensic odontology and Cheiloscopy among the study participants (n=320) n: number, k: knowledge question

Item	Question	Responses	n	%
K1	Forensic odontology is a branch of dentistry	Yes	251	78.4%
		No	69	21.6%
K2	Forensic odontology useful in identifying criminals and the dead people	Yes	231	72.2%
		No	26	8.1%
		Don’t know	63	19.7%
K3	Formal training related to forensic odontology	Yes	38	11.9%
		No	282	88.1%
K4	Forensic odontology as part of your curriculum or course outline	Yes	66	20.6%
		No	254	79.4%
K5	Saudi Arabia has very limited resources/equipment to study forensic science	Yes	234	73.1%
		No	86	26.9%
K6	Identification of a deceased person’s age and gender in mass disasters like fire, stampede, and accidents	Reconstruct the fragmented deceased body	27	8.4%
		Dental records	151	47.2%
		Fingerprints	20	6.3%
		Lip prints	14	4.4%
		Don’t know	108	33.8%
K7	The study of lip prints in forensic dentistry is	Lipology	32	10.0%
		Cheiloscopy	117	36.6%
		Dermatoglyphics	19	5.9%
		don’t know	152	47.5%
K8	The anatomical landmarks of the lip	Yes	196	61.3%
		No	124	38.8%
K9	Lips have furrows and grooves	Yes	205	64.1%
		No	115	35.9%
K10	Most commonly used classification of lip prints	Yes	59	18.4%
		No	261	81.6%
K11	There are five lip groove patterns according to Tsuchihashi and Suzuki classification	Yes	64	20.0%
		No	256	80.0%
K12	Most predominant groove pattern	Type I	26	8.1%
		Type II	16	5.0%
		Type III	16	5.0%
		Type IV	5	1.6%
		Type V	6	1.9%
		Don’t know	251	78.4%
K13	Type III is the most common lip groove pattern among males	Yes	50	15.6%
		No	270	84.4%
K14	Type I is the most common lip groove pattern among females	Yes	72	22.5%
		No	248	77.5%
K15	Methods of lip prints collection	Yes	68	21.3%
		No	252	78.8%

**Figure 1:**
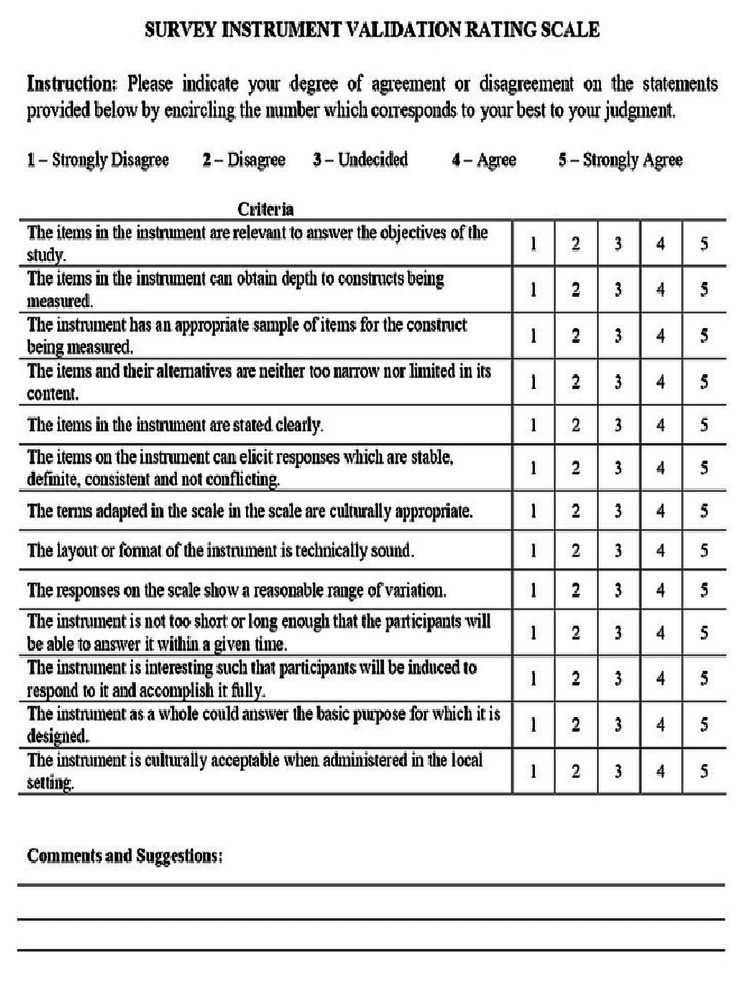
Survey instrument validation rating scale

As for the validation of the questionnaire, the Survey instrument validation rating scale (Figure 1) was employed, as can be seen below.

The criteria for evaluating survey questionnaires provided by Good and Scates, the definition of content validity provided by Polit and Beck, and the Chavez and Canino criteria for the Cultural Equivalency Model for Translating and Adapting Instruments were used to develop the statements of this survey instrument validation rating scale. It also considers Johnson's definition of facial validation. The Institutional Review Board of REU had approved the validation tool at the time of their review of this investigation.

The collected data was analyzed using IBM Corp. Released 2017. IBM SPSS Statistics for Windows, Version 25.0. Armonk, NY: IBM Corp. For the categorical variables, descriptive statistics of frequency distribution and percentages were calculated using non-parametric methods. The Chi-square and Fisher's exact tests were applied to find out the association between the characteristics of the study participants and their knowledge (as obtained through their study/course curriculum) and awareness of forensic odontology. Statistical significance was determined at a p-value of 0.05.

## Results

In the present study, a total of 400 questionnaires were distributed to the study participants using the Fisher method (as mentioned in the materials and methods section). However, 80 individuals did not complete the questionnaire due to time constraints and, thereby, were dropped from the final data analysis; this could be determined from the fact that, as mentioned earlier, the forms were distributed both offline and online (Google forms) mode so the number of questionnaires in circulation could be easily tracked. A final response rate of 80.8% was obtained.

Distribution of study participants

A total of 320 dental professionals completed the questionnaire, including 84 undergraduates, 60 graduates, 58 postgraduate dental students, and 118 general dental practitioners who were a part of the institutions (in various capacities of employment) that had consented to join our investigation. Demographic data revealed that the majority of participants were males (177, 55.3%). The majority of them were aged between 23 and 27 years. The majority, 118 (36.9.%) of the participants were general dental practitioners (GDP), followed by undergraduates, 84 (26.3%), graduates (who had just graduated from a dental college/institution and were not practicing or undergoing any internship), 60 (8.8%), and postgraduates, 58 (18.1%). Among four universities that participated in the present study, 90 (28.1%) individuals were from Riyadh Elm University (REU), Riyadh, 70 (21.9%) were from King Khalid University (KKU), Abha, 65 (20.3%) were from King Saud Bin Abdulaziz University for Health Sciences (KSAU-HS), Riyadh, and 95 (29.7%) belonged to Imam Abdurrahman bin Faisal University (IAFU), Dammam (Table 2).

**Table 2:** Demographic characteristics of the study participants N: number

Characteristics		n	%
Age	18-22	82	25.6%
	23-27	125	39.1%
	28-32	113	35.3%
	Total	320	100.0%
Gender	Male	177	55.3%
	Female	143	44.7%
	Total	320	100.0%
Education	Undergraduates	84	26.3%
	Graduates	60	18.8%
	Postgraduates	58	18.1%
	General dental practitioners	118	36.9%
	Total	320	100.0%
University	REU	90	28.1%
	KSAU-HS	65	20.3%
	KKU	70	21.9%
	IAFU	95	29.7%
	Total	320	100.0%

Knowledge of forensic odontology and cheiloscopy among the study participants

The details of the questionnaire, as well as the scoring criterion, are mentioned in Table 1. About 78.4% of the participants’ replied "Yes" to the knowledge of forensic dentistry, and 72.2% agreed that forensic odontology is useful for the identification of criminals and dead people. About 88.1% reported that they had no formal training in this field, and 79.4% agreed that forensic odontology was not included in the dental curriculum or the course outline. The majority of them (73.1%) agreed with the fact that Saudi Arabia has limited resources in the field of forensic dentistry. Most (47.2%) of the participants reported that dental records help in the identification of a deceased person’s age and gender in mass disasters. The study of lip prints in forensic dentistry is called "Cheiloscopy," and was replied correctly by only 36.6%, whereas 47.5% answered, "Don’t know," even though (61.3%) and (64.1%) were aware of the anatomical landmarks and grooves and furrows of the lips.

Overall, a majority (81.6%) of the participants reported that they were not aware of the classification of lip prints, and about 80% were not aware of a specific classification of lip prints by Tsuchihashi and Suzuki, and 78.4% reported: "Don’t know." Only 8.1% replied that Type 1 is the most predominant lip groove pattern.

Type III is the most common lip groove pattern in males and was reported correctly by 15.6%, whereas a majority (84.4%) replied "Don’t know." Type I pattern is the most common in females and was agreed upon by 22.5% of all participants, whereas 77.5% reported "No." Regarding lip print collection methods, 78.8% of the participants didn’t know about them (Table 1).

Overall, the majority of the participants had adequate knowledge of forensic odontology. However, their knowledge of lip prints was inadequate.

Awareness of cheiloscopy among the study participants

About 43.1% of the participants were aware of the significance of lip print patterns in gender identification, whereas 56.9% were not aware of it. The majority (55%) were unaware that lip prints are unique and will differ between individuals. The significance of lip prints in forensic odontology, as an investigation tool for the identification of humans involved in the crime or victims, was replied to correctly by half of them, and another half reported that they were unaware of it. The use of lip prints as biomarkers in genetic diseases was known to 34.4%, and a majority (65.6%) were not aware. A majority (78.4%) of the participants were not aware that lip prints were used for screening type II diabetes mellitus (Table 3).

**Table 3:** Awareness of cheiloscopy among the study participants (n=320) N: Number A: Awareness questions

Item	Questions	Responses	N	%
A1	Significance of lip print pattern in gender identification	Yes	138	43.1%
		No	182	56.9%
A2	Lip prints are unique and permanent for each individual	Yes	144	45.0%
		No	176	55.0%
A3	Lip prints can be used as forensic investigation tool in for identification of humans based on lip traces	Yes	160	50.0%
		No	160	50.0%
A4	Lip prints are used as biomarkers in genetic diseases	Yes	110	34.4%
		No	210	65.6%
A5	Lip prints are used as screening tool for detecting type II diabetes	Yes	69	21.6%
		No	251	78.4%

Overall, the majority of the participants lacked awareness of cheiloscopy as evidenced by the responses that can be seen in Table 3. Comparison and assessment of knowledge of forensic odontology and cheiloscopy among different genders, educational levels, and universities. Questions from K1-K6 were knowledge-based questions on forensic odontology. Questions from K7-K15 were knowledge-based questions on cheiloscopy (Table 4). The age and gender, as represented in Table 4, were important to this investigation because cheiloscopy and its various applications are specific skills and domains of knowledge that could be dependent upon whether the individual has been exposed to it in their daily curriculum/practice since, as per a dental point of view, cheiloscopic investigations are not a usual occurrence in dental scenarios (whether be a clinic or as part of their study course). As for the comparisons between an undergraduate and a general practitioner, we based our questionnaire on a premise that cheiloscopy and its related applications could be encountered by an individual invested in dental practice, whether it be through his/her course curriculum or clinic/hospital (Table 4).

**Table 4:** Comparison of knowledge of cheiloscopy among age, gender, educational levels and universities of the study participants N: number, K: Knowledge questions

Items		Age (%)				Gender (%)			Education (%)					University (%)				
		18-22	23-27	28-32	p	Male	Female	p	Undergraduates	Graduates	Postgraduates	General dental practitioners	p	REU	KSU-HS	KKU	IAFU	P
K1	Yes	70.70	85.60	76.10	0.03	71.80	86.70	0.001	78.60	86.70	75.90	75.40	0.353	66.70	78.50	87.10	83.20	0.008
	No	29.30	14.40	23.90		28.20	13.30		21.40	13.30	24.10	24.60		33.30	21.50	12.90	16.80	
K2	Yes	64.60	85.60	62.80	0.00	60.50	86.70	0.000	71.40	91.70	65.50	66.10	0.013	64.40	76.90	71.40	76.80	0.568
	No	12.20	2.40	11.50		13.00	2.10		9.50	0.00	12.10	9.30		11.10	6.20	7.10	7.40	
	Dont know	23.20	12.00	25.70		26.60	11.20		19.00	8.30	22.40	24.60		24.40	16.90	21.40	15.80	
K3	Yes	14.60	8.00	14.20	0.23	15.30	7.70	0.038	11.90	6.70	24.10	8.50	0.011	22.20	10.80	11.40	3.20	0.001
	No	85.40	92.00	85.80		84.70	92.30		88.10	93.30	75.90	91.50		77.80	89.20	88.60	96.80	
K4	Yes	22.00	23.20	16.80	0.45	24.90	15.40	0.037	19.00	35.00	24.10	12.70	0.005	28.90	9.20	17.10	23.20	0.020
	No	78.00	76.80	83.20		75.10	84.60		81.00	65.00	75.90	87.30		71.10	90.80	82.90	76.80	
K5	Yes	69.50	84.00	63.70	0.00	64.40	83.90	0.000	82.10	85.00	63.80	65.30	0.003	68.90	81.50	77.10	68.40	0.186
	No	30.50	16.00	36.30		35.60	16.10		17.90	15.00	36.20	34.70		31.10	18.50	22.90	31.60	
K6	Reconstruct the fragmented deceased body	17.10	6.40	4.40	0.00	8.50	8.40	0.253	11.90	13.30	5.20	5.10	0.000	11.10	9.20	10.00	4.20	0.000
	Dental records	40.20	64.00	33.60		48.00	46.20		44.00	65.00	51.70	38.10		51.10	52.30	51.40	36.80	
	Fingerprints	11.00	4.00	5.30		8.50	3.50		9.50	1.70	17.20	0.80		11.10	15.40	0.00	0.00	
	Lip prints	3.70	5.60	3.50		5.10	3.50		7.10	3.30	5.20	2.50		6.70	4.60	4.30	2.10	
	Don’t know	28.00	20.00	53.10		29.90	38.50		27.40	16.70	20.70	53.40		20.00	18.50	34.30	56.80	
K7	Lipology	18.30	10.40	3.50	0.00	10.70	9.10	0.061	15.50	11.70	8.60	5.90	0.000	13.30	6.20	15.70	5.30	0.000
	Cheiloscopy	37.80	43.20	28.30		34.50	39.20		41.70	46.70	44.80	23.70		36.70	58.50	34.30	23.20	
	Dermatoglyphics	13.40	0.80	6.20		9.00	2.10		6.00	1.70	13.80	4.20		10.00	7.70	7.10	0.00	
	don’t know	30.50	45.60	61.90		45.80	49.70		36.90	40.00	32.80	66.10		40.00	27.70	42.90	71.60	
K8	Yes	58.50	81.60	40.70	0.00	57.60	65.70	0.139	72.60	66.70	63.80	49.20	0.005	61.10	75.40	84.30	34.70	0.000
	No	41.50	18.40	59.30		42.40	34.30		27.40	33.30	36.20	50.80		38.90	24.60	15.70	65.30	
K9	Yes	64.60	85.60	39.80	0.00	60.50	68.50	0.134	77.40	85.00	60.30	45.80	0.000	58.90	84.60	77.10	45.30	0.000
	No	35.40	14.40	60.20		39.50	31.50		22.60	15.00	39.70	54.20		41.10	15.40	22.90	54.70	
K10	Yes	23.20	14.40	19.50	0.26	20.90	15.40	0.206	21.40	15.00	36.20	9.30	0.000	33.30	20.00	18.60	3.20	0.000
	No	76.80	85.60	80.50		79.10	84.60		78.60	85.00	63.80	90.70		66.70	80.00	81.40	96.80	
K11	Yes	23.20	18.40	19.50	0.69	23.70	15.40	0.064	20.20	16.70	36.20	13.60	0.005	31.10	23.10	22.90	5.30	0.000
	No	76.80	81.60	80.50		76.30	84.60		79.80	83.30	63.80	86.40		68.90	76.90	77.10	94.70	
K12	Type I	9.80	4.80	10.60	0.00	12.40	2.80	0.007	4.80	6.70	20.70	5.10	0.055	17.80	7.70	7.10	0.00	0.000
	Type II	11.00	2.40	3.50		6.20	3.50		8.30	6.70	3.40	2.50		11.10	6.20	1.40	1.10	
	Type III	8.50	4.80	2.70		5.10	4.90		3.60	6.70	6.90	4.20		7.80	3.10	8.60	1.10	
	Type IV	4.90	0.80	0.00		2.30	0.70		2.40	0.00	1.70	1.70		0.00	3.10	4.30	0.00	
	Type V	2.40	0.80	2.70		2.80	0.70		2.40	0.00	3.40	1.70		2.20	1.50	2.90	1.10	
	Dont know	63.40	86.40	80.50		71.20	87.40		78.60	80.00	63.80	84.70		61.10	78.50	75.70	96.80	
K13	Yes	20.70	13.60	14.20	0.33	21.50	8.40	0.001	15.50	10.00	24.10	14.40	0.190	26.70	12.30	20.00	4.20	0.000
	No	79.30	86.40	85.80		78.50	91.60		84.50	90.00	75.90	85.60		73.30	87.70	80.00	95.80	
K14	Yes	25.60	12.00	31.90	0.00	19.20	26.60	0.117	19.00	10.00	20.70	32.20	0.006	23.30	15.40	21.40	27.40	0.353
	No	74.40	88.00	68.10		80.80	73.40		81.00	90.00	79.30	67.80		76.70	84.60	78.60	72.60	
K15	Yes	29.30	16.00	21.20	0.07	25.40	16.10	0.042	20.20	15.00	29.30	21.20	0.295	23.30	35.40	21.40	9.50	0.001
	No	70.70	84	78.8		74.60	83.9		79.80	85.00	70.70	78.8		76.70	64.60	78.60	90.50	

A majority (86.7%) of the female participants were more aware of forensic odontology as a branch of dentistry than males. At p 0.05, the difference is statistically significant. The majority of the graduates (86.7%) replied "Yes" more often than others. However, most of the general dental practitioners (24.6%) were not aware of it. About 87.1% of the KKU students were aware that forensic odontology is a branch of dentistry, whereas 33.3% of the REU students were not aware. Forensic odontology is useful for the identification of criminals and deceased people and was known only to 60.5% of male participants. About 91.7% of the graduate dental students replied, "Yes." However, only 65.5% of postgraduates reported affirmative, and this was statistically significant at p 0.05. Most (76.9%) of KSAU-HS students answered more correctly than students at the other three universities.

Forensic odontology as part of the curriculum or course outline. For this item, the majority of the participants answered "No." Only 20.6% of undergraduate students replied "Yes," whereas 80% replied "No." About 83.9% of the female participants agreed that Saudi Arabia has limited resources to study forensic sciences. However, 35.6% of males disagreed with them. Eighty-five percent of graduate dental students agreed, whereas 36.2% of postgraduate students disagreed about Saudi Arabia having limited resources in forensic dentistry. A majority (81.5%) of students from KSAU-HS agreed with others. However, 31.6% of IAFU students disagreed with other universities.

Sixty-five percent of graduates replied correctly, followed by postgraduates (51.7%) about the importance of forensic sciences-dental records in the identification of deceased people’s age and gender in mass disasters. About 53.4% of general dental practitioners were not aware of it. Cheiloscopy, the study of lip prints, was known by 36.6% of all the participants. The majority of the participants (63.4%) were not aware. Female (39.2%) participants and postgraduate (46.7%) students replied correctly, whereas the majority (66.1%) of the general dental practitioners and 71.6% of IAFU students were not aware. About 58.5% of KSAU-HS students replied correctly. The anatomical landmarks and grooves and furrows of the lips were known to the majority (61.3% and 64.1%) of the participants. The majority of female (65.7, 68.5%%) participants were more aware of anatomical landmarks, lip grooves, and furrows than their male counterparts. Most of the undergraduates (72.6% and 77.4%) replied "Yes" to anatomical landmarks and lip grooves and furrows. However, the majority (50.8%, or 54.2%) of general dental practitioners were not aware. Among university students, KKU (84.3%, 77.1%), followed by KSAU-HS (75.4%, 84.6%), were aware. However, students of IAFU (65.3% and 54.7%) were not aware of the anatomical landmarks of the lips.

The lip print classification given by Tsuchihashi and Suzuki was not known to the majority (81.6%) of the participants. About 20.9% more males were aware of it than females. A majority of general dental practitioners (90.7%) were not aware of it. However, 36.2% of postgraduates were aware. Most of the students from the four universities were not aware. However, a small percentage (33.3%) of students from REU were aware of it. The presence of five lip print patterns was unknown to 80% of all the participants. However, 23.7% of male participants and 36.2% of postgraduate students were aware, and 31.1% of students in REU were able to answer correctly compared to other university students. Students of IAFU, the majority (94.7%) of them were not aware of it. The knowledge about the most predominant lip print pattern, Type I, was correctly answered by only 8.1% of all the participants, whereas 78.4% replied that they were not aware of it. The majority (87.4%) of female participants reported "Don’t know" compared to their male counterparts. Most of the postgraduate students (20.7%) replied correctly across all education levels. About 96.8% of general dental practitioners were not aware. Among universities, the majority of REU students (17.8%) answered correctly more often than other university students. The knowledge of IAFU students was low because a majority (96.8%) of them were not aware. Type III, the most common lip print pattern among males, was correctly replied to by only 15.6% of all the participants. Whereas the majority (84.4%) of the participants reported "No." Males (21.5%) replied more correctly than females, and the majority of postgraduates (24.1%) answered "Yes" compared to other study participants. Among all universities, the students of REU (26.7%) answered correctly and were aware of it. However, 95.8% of IAFU students were not aware. Type I, which is the most common lip print pattern among females, was known only to 22.5% of all participants, whereas 77.5% of them were not aware. Most of the male (80.8%) participants were not aware of it, whereas 26.6% of females had knowledge of it. Among educational levels, postgraduates (20.7%) reported "Yes" compared to others. However, the majority of general dental practitioners (32.2%) were aware of it. About 23.3% of REU students were aware that the most common lip print for females is Type I, followed by other university students. Overall, 78.8 % replied "No" to the methods of collection of lip prints, and only 21.3% replied "Yes" among all participants. The majority of males (25.4%) have more knowledge about it than females. About 29.3% of postgraduates have more knowledge about it than others, and 35.4% of KSAU-HS students replied "Yes," followed by REU students (23.3%).

Comparison and assessment of awareness of cheiloscopy among different genders, educational levels, and universities.

Questions from A1-A5 were awareness-based questions on cheiloscopy (Table 5).

**Table 5:** Comparison of awareness of cheiloscopy among different age groups, gender, educational levels and universities of the study participants N: Number, A: Awareness questions

Items		Age				Gender			Educational levels					University				
		18-22	23-27	28-32	p	Male	Female	p	undergraduates	Graduates	Post graduates	General dental practitioners (GDP)	p	REU	KSU-HS	KKU	IAFU	p
A1	Yes	40.2%	33.6%	55.8%	.002*	41.2%	45.5%	0.449	33.3%	31.7%	56.9%	49.2%	.005*	43.3%	49.2%	42.9%	38.9%	0.644
	No	59.8%	66.4%	44.2%		58.8%	54.5%		66.7%	68.3%	43.1%	50.8%		56.7%	50.8%	57.1%	61.1%	
A2	Yes	52.4%	45.6%	38.9%	0.171	44.6%	45.5%	0.883	50.0%	40.0%	60.3%	36.4%	.015*	41.1%	60.0%	51.4%	33.7%	.006*
	No	47.6%	54.4%	61.1%		55.4%	54.5%		50.0%	60.0%	39.7%	63.6%		58.9%	40.0%	48.6%	66.3%	
A3	Yes	52.4%	36.8%	62.8%	.000*	47.5%	53.1%	0.312	42.9%	36.7%	60.3%	56.8%	.014*	44.4%	60.0%	45.7%	51.6%	0.229
	No	47.6%	63.2%	37.2%		52.5%	46.9%		57.1%	63.3%	39.7%	43.2%		55.6%	40.0%	54.3%	48.4%	
A4	Yes	31.7%	28.0%	43.4%	.038*	31.1%	38.5%	0.167	31.0%	26.7%	29.3%	43.2%	0.080	34.4%	21.5%	30.0%	46.3%	.010*
	No	68.3%	72.0%	56.6%		68.9%	61.5%		69.0%	73.3%	70.7%	56.8%		65.6%	78.5%	70.0%	53.7%	
A5	Yes	32.9%	13.6%	22.1%	.004*	28.2%	13.3%	.001*	25.0%	16.7%	32.8%	16.1%	.050*	40.0%	20.0%	22.9%	4.2%	.000*
	No	67.1%	86.4%	77.9%		71.8%	86.7%		75.0%	83.3%	67.2%	83.9%		60.0%	80.0%	77.1%	95.8%	

Awareness of cheiloscopy in gender identification was not known to a majority (56.9%) of the participants. A majority (55.8%) of participants aged between 28 and 32 years replied correctly. At p 0.005, the difference is statistically significant. The majority of males (58.8%) were not aware. Among educational levels, most (56.9%) of the postgraduate students reported "Yes," and 50.8% of general dental practitioners answered " No". At p 0.005, the difference is statistically significant. Among the universities, the majority of KSAU-HS (49.2%), followed by REU (43.3%), were aware, whereas the participants from IAFU (61.1%) replied "No" as they were not aware. About 45.5% of females and 52.4% of 18 to 22-year-old participants replied correctly to the question about the uniqueness of lip prints for each individual. About 60.3% of postgraduates reported "Yes," and 63.6% of general dental practitioners replied "No" as they were not aware of it, statistically significant at p=0.005. Sixty percent of KSAU-HS students correctly answered the question. At p 0.005, the difference is statistically significant.

Lip prints as investigation tools in the identification of humans based on lip traces were answered correctly by 62.8% of the participants aged 28-32 years. At p 0.005, the difference is statistically significant. Among females, 53.1% of females and about 60.3% of postgraduates replied "Yes," whereas 63.3% of graduates did not agree. At p 0.005, the difference is statistically significant. KSAU-HS students were more aware compared to others who disagreed. Awareness of lip prints as biomarkers in genetic diseases was known to only 34.4% of all participants. Among the age groups, a majority (43.4%) of 28 to 32-year-old aged participants, about 38.5% of females, and 43.2% of general dental practitioners and students of IAFU (46.3%) were aware. However, a majority (73.3%) of graduates and KSAU-HS students (78.5%) were not aware. At p 0.005, the difference is statistically significant.

The lip prints used for screening and detecting type II diabetes mellitus were replied to by 32.9% of participants aged 18-22 years correctly. A majority (86.7%) of females were not aware. At p 0.005, the difference is statistically significant. About 32.8% of postgraduates and 40% of REU students were aware, while the majority (95.8%) of IAFU students were not. At p 0.005, the difference is statistically significant. 

## Discussion

Forensic odontology has played a key role in the identification of people in mass disasters. The different methods used in forensic odontology include rugoscopy, cheiloscopy, bite marks, tooth prints, radiographs, photographic studies, and molecular methods. The external surface of the lip has many elevations and depressions that form the characteristic patterns referred to as "lip prints. At the crime scene, lip prints can be obtained from clothing, cups, glasses, cigarettes, windows, and doors. The different lip print patterns identified include vertical, intersected, branched, reticular, and undetermined. The anatomical landmarks of the lip include the chelion (the lateral most point in the mouth opening), the stomian (the contact of upper and lower lips in the mid-sagittal plane), the labrale superius, and the labrale inferius (the highest and lowest points of upper and lower lip margins in the mid-sagittal plane, respectively). The lip prints have to be obtained within 24 hours of the time of death to prevent erroneous data that may occur due to postmortem lip alterations. In the closed-mouth position, the lip prints obtained will be well-defined grooves, whereas, in the open-mouth position, they are ill-defined and very difficult to interpret [8,9]. Lip prints are unique to an individual, and no two individuals have the same lip prints, even if they are from the same family [8,9].

In the forensic dentistry studies conducted by Rahman J et al. [10] and Nagarajappa R et al. [11] among dental professionals in India, the majority of postgraduate (MDS) dental students (86.5% and 81.5%), followed by undergraduates (77.5% and 59.1%), had adequate knowledge of lip print patterns in human identification. This finding is similar to the present study in which a majority of postgraduates (60.3%) replied correctly than undergraduates (42.9%). Awareness of lip print identification among dental practitioners was replied to as "Yes" by 88.10% of the participants in a study conducted by Bhakhri S et al. [12]. This is in contrast to the present study, in which 50% of the participants disagreed [13]. The study of lip prints called cheiloscopy was answered correctly by 63% of all participants [14], whereas only 36.6% of participants responded "Yes" in the present study, and the majority were postgraduates (46.7%).

The present study is consistent with other studies [7, 14] about limited resources to study forensic sciences in Saudi Arabia and other countries. In a study conducted in Saudi Arabia among dental students by Abdul NS et al. [7] and other studies [15], the majority (77.5%) of postgraduates than undergraduates (72%) agreed that forensic odontology helps in the identification of a deceased person's age and gender in mass disasters using dental records. This finding is similar to the present study, which is in line with these studies and reported that the majority (51.7%) of the postgraduates agreed compared to undergraduates (44%).

No formal training in forensic odontology was agreed upon by most of the studies [2,16-18]. About 97.5% agreed that forensic odontology is not a part of the curriculum [2], which is similar to the present study, in which 79.4% of the participants agreed. Duraimurugan S et al. [19], in their study on forensic dentistry among dental students and practitioners in India, revealed that 50.8% were aware of cheiloscopy, and 35.4% replied, "Don’t know." However, our study findings contradict these studies, in which the majority (47.5%) answered, "Don’t know," whereas only 36.6% replied, "Yes." The uniqueness of lip prints was known to 60% of the participants, and 19% were not aware [7], which is in contrast to the present study, in which 55% replied that they were not aware, and only 45% were aware. In terms of knowledge of different types of lip print patterns, 50% of the participants were aware; this statement is in contrast to the present study, in which the majority, 80%, were not aware, and only 20% were aware. One of the studies reported that lip patterns are different for males and females. Type 1 is the most common lip print in females and Type 4 and 5 in males [8,20]. In the present study, only a few participants (43.1%) agreed that lip prints are helpful for gender identification and no two individuals will have similar lip prints, whereas the majority of the participants were not aware. Diabetes is a global disease, and Type 2 diabetes mellitus is more common and is genetically influenced. The diagnosis of this condition at an early stage will improve the lifestyle and health of an individual; therefore, lip prints may serve as biomarkers in screening for diabetes mellitus. In a few studies, it was found that Type II (branched grooves) and IV (reticular) lip print patterns are common among Type 2 diabetics and Type I (vertical grooves throughout the lip) in controls [10,21]. In the present study, knowledge about lip prints as screening tools for detecting Type II diabetes mellitus was not known to the majority (78.4%) of the participants, whereas only 21.6% were aware.

Our study's strengths include the fact that, whereas lip prints and forensic odontology have been extensively studied in other areas of the world, research on these topics has been scarce or nonexistent in Saudi Arabia. To evaluate the knowledge and awareness of cheiloscopy, which was also the main objective of our investigation, we targeted undergraduate, graduate, and postgraduate dental students as well as general dentistry practitioners from several regional institutions.

Regarding limitations, this study is restricted to a small number of universities and does not adequately represent the entire Saudi Arabian nation. Additionally, research relying on questionnaires typically is unable to provide reliable descriptions of a field of study like cheiloscopy. Moreover, we would also like to mention the fact that the sample size for our investigation is somewhat limited in comparison to other studies (even related to different fields of research) as far as questionnaire-based studies are concerned. Additionally, there is very little research on dental professionals' knowledge of new developments in the field of cheiloscopy, which made it difficult to thoroughly compare our results with those of other reliable studies. Therefore, further studies should be done in multiple regions or provinces involving larger populations of Saudi Arabia.

## Conclusions

Overall, as per the initial objective that we set out to establish through the means of this study, the knowledge and awareness of cheiloscopy among all study participants was inadequate (as evident by the responses recorded in the questionnaire). As would be expected, postgraduate dentistry students showed a greater understanding of cheiloscopy than undergraduates and graduates. In contrast to postgraduate students, general dentistry practitioners lacked cheiloscopy knowledge and awareness. Students from Riyadh Elm University among the universities showed appropriate understanding but were less familiar with cheiloscopy than other university students. If required steps are taken to include forensic odontology in the dentistry curriculum in Saudi Arabia, this obvious knowledge gap, particularly among undergraduate students and general practitioners, can be reduced.
